# Serial Fecal Microbiota Infusions via Colonoscopy for Active Ulcerative Colitis: A Feasibility, Safety, and Translational Monocentric Italian Study

**DOI:** 10.3390/microorganisms11102536

**Published:** 2023-10-11

**Authors:** Loris Riccardo Lopetuso, Lucrezia Laterza, Valentina Petito, Silvia Pecere, Gianluca Quaranta, Federica Del Chierico, Pierluigi Puca, Elisa Schiavoni, Daniele Napolitano, Andrea Poscia, Gianluca Ianiro, Daniela Pugliese, Lorenza Putignani, Maurizio Sanguinetti, Alessandro Armuzzi, Luca Masucci, Antonio Gasbarrini, Giovanni Cammarota, Franco Scaldaferri

**Affiliations:** 1 IBD Unit, CEMAD, Digestive Diseases Center, Medicina Interna e Gastroenterologia, Dipartimento di Scienze Mediche e Chirurgiche, Fondazione Policlinico Universitario “A. Gemelli” IRCCS, L. Go A. Gemelli 8, 00168 Rome, Italy; lopetusoloris@gmail.com (L.R.L.); lucrezia.laterza@policlinicogemelli.it (L.L.); valentina.petito@unicatt.it (V.P.); silvia.pecere@policlinicogemelli.it (S.P.); pgpuca@gmail.com (P.P.); elisa.schiavoni@policlinicogemelli.it (E.S.); daniele.napolitano@policlinicogemilli.it (D.N.); daniela.pugliese@policlinicogemelli.it (D.P.); antonio.gasbarrini@unicatt.it (A.G.); 2 Department of Medicine and Ageing Sciences, “G. d’Annunzio” University of Chieti-Pescara, 66100 Chieti, Italy; 3 Center for Advanced Studies and Technology (CAST), “G. d’Annunzio” University of Chieti-Pescara, 66100 Chieti, Italy; 4 Dipartimento di Medicina e Chirurgia Traslazionale, Università Cattolica del Sacro Cuore, L. Go F. Vito 1, 00168 Rome, Italy; gianluca.ianiro@unicatt.it (G.I.); giovanni.cammarota@unicatt.it (G.C.); 5 Microbiology Unit, Fondazione Policlinico Universitario ‘A. Gemelli’ IRCCS, Università Cattolica del Sacro Cuore, 00168 Rome, Italy; gianluca.quaranta@policlinicogemelli.it (G.Q.); maurizio.sanguinetti@policlinicogemelli.it (M.S.); luca.masucci@policlinicogemelli.it (L.M.); 6 Unit of Human Microbiome, Bambino Gesù Children’s Hospital, IRCCS, 00168 Rome, Italy; federica.delchierico@opbg.net; 7 Section of Hygiene, University Department of Life Sciences and Public Health, Università Cattolica del Sacro Cuore, 00168 Roma, Italy; andreaposcia@yahoo.com; 8 UOC ISP Prevention and Surveillance of Infectious and Chronic Diseases, Department of Prevention, Local Health Authority (ASUR-AV2), 60035 Jesi, Italy; 9 UOC di Gastroenterologia, Fondazione Policlinico Universitario “A. Gemelli” IRCCS, L. Go A. Gemelli 8, 00168 Rome, Italy; 10 Unit of Microbiomics and Unit of Human Microbiome, Bambino Gesù Children’s Hospital, IRCCS, 00168 Rome, Italy; lorenza.putignani@opbg.net; 11 IBD Center, IRCCS Humanitas Research Hospital, Rozzano, 20089 Milan, Italy; alessandro.armuzzi@unicatt.it

**Keywords:** fecal microbiota transplantation, ulcerative colitis, serial infusions

## Abstract

The effectiveness of fecal microbiota transplantation (FMT) in ulcerative colitis (UC) remains unclear. This study aimed to investigate the feasibility and effectiveness of serial fecal infusions via colonoscopy in patients with active UC. Subjects with mild-to-moderate UC received three consecutive fecal infusions via colonoscopy. A control population with the same baseline features receiving Infliximab treatment was enrolled. Adverse events and clinical, endoscopic, and microbial outcomes were investigated. Nineteen patients with mildly-to-moderately active UC were enrolled. Clinical response was obtained in six patients at week 2, in eight at week 6, and in nine at week 12. Clinical response was maintained in eight patients at week 24. Endoscopic remission at week 12 was reached in six patients. In the control population, 13/19 patients achieved clinical response at week 6, and 10/19 patients maintained clinical response after 6 months. Microbiota richness was higher in responders compared with the non-responders. *Peptostreptococcus*, *Lactobacillus*, and *Veillonella* were higher in non-responders, while *Parabacteroides*, *Bacteroides*, *Faecalibacterium*, and *Akkermansia* were higher in responders at all timepoints. Serial FMT infusions appear to be feasible, safe, and effective in UC patients, with a potential role in inducing and maintaining clinical response. Specific bacteria predict the response to FMT.

## 1. Introduction

Inflammatory bowel diseases (IBDs), encompassing Crohn’s disease (CD) and ulcerative colitis (UC), are chronic, relapsing inflammatory disorders of the digestive tract, resulting from a loss of homeostasis between the intestinal immune system and the gut microbiota in genetically predisposed individuals [1]. Inappropriate mucosal immune responses, due to dysregulation of tolerance to intestinal microbiota or disruption of the epithelial barrier separating microorganisms from underlying tissues, may contribute to the development or perpetuation of IBD.

Increasing evidence suggests that the imbalance of gut microbiota, the so-called “dysbiosis”, is one of the most influencing environmental factors that could promote the development of UC, as the interaction of this altered microbiota with the human host could trigger and foster the immune alterations that are associated with UC [2].

Therefore, the enthusiasm toward the use of therapeutic manipulation of gut microbiota as a potential treatment for UC, both from patients and physicians, has risen in recent years. However, this concept has still hardly been translated into clinical practice, as to date, only a few probiotics have shown some effectiveness in inducing or maintaining remission in patients with mild-to-moderate UC [3].

Fecal microbiota transplantation (FMT) is the infusion of feces from healthy donors into the gut of a recipient to treat a disease associated with the impairment of gut microbiota. FMT is clearly recognized as a highly effective treatment option for recurrent *Clostridioides difficile* infection (CDI), as shown by several randomized clinical trials [4,5,6,7,8] and meta-analyses [9,10,11]. Therefore, international guidelines have included FMT as a treatment option for this condition [12,13].

After being successfully used for CDI, FMT was also investigated in patients with UC, first in non-randomized [14,15,16] studies, and then in randomized clinical trials [17,18,19,20,21,22], with promising results but substantial differences in FMT working protocols. Our study aims to investigate feasibility and effectiveness of FMT, performed by colonoscopies, in patients affected by mildly-to-moderately active UC.

## 2. Materials and Methods

### 2.1. Study Design and Patients’ Population

This is an open-label pilot study using a prospective cohort, performed at the “Fondazione Policlinico Universitario Agostino Gemelli”, a tertiary academic hospital set in Rome, Italy, approved by the local internal Ethics Committee (ID 100358) and conducted according to the principles expressed in the Declaration of Helsinki. All participants provided written informed consent (PROT CE/10930/15).

Eligible patients were 18 years or older with mildly-to-moderately active UC defined as a partial Mayo score equal to or more than 4 and an endoscopic Mayo score equal to or less than 1, with an upper limit Mayo score equal to or more than 11. Concomitant medicines for UC, such as mesalamine, glucocorticoids, and immunosuppressive therapy (e.g., azathioprine), were allowed at stable doses for a designated period of time: 5-ASA or sulfasalazine at a stable dose for at least 2 weeks prior to starting FMT treatment and during the study treatment period; and methotrexate (up to 15 mg/week subcutaneously), azathioprine (up to 2.5 mg/kg/die), or 6-mercaptopurine (up to 2 mg/kg/die) at a stable dose for at least 8 weeks.

Patients were excluded if they had taken antibiotics or probiotics in the last 30 days, had concomitant infection from *C. difficile* or another enteric pathogen, or had a disease severity requiring hospitalization; furthermore, they were excluded if they were pregnant or were unable to give informed consent.

After a screening period lasting two weeks, the patients underwent FMT including repeated fecal infusions through three colonoscopies: at baseline (time 0), after 2 weeks (time 1), and after 6 weeks (time 2). Patients were followed up for safety and efficacy outcomes at week 12 via clinical and endoscopic assessment (sigmoidoscopy) and at week 24. These timepoints are consistent with those commonly used in clinical practice for biologic therapy, especially for Infliximab, that we hereby use as a comparison.

Patients were given a symptom diary to be filled in every day by themselves or by family members on their behalf and checked by the medical and nursing staff. Patients (or family members) were also questioned about stool frequency and consistency, medication use, and adverse events in the follow-up period.

In addition, during the same period, we enrolled a cohort of patients with the same eligibility criteria, who would receive treatment with Infliximab at a dose of 5 mg/kg at the standard-of-care timepoints: at baseline (time 0), after 2 weeks (time 1), after 6 weeks (time 2), and every 8 weeks subsequently.

### 2.2. Aims of the Study and Outcomes Measures

In this study, we aimed to evaluate the feasibility and effectiveness of serial fecal infusions via colonoscopy in patients with mildly-to-moderately active UC. The primary outcome was the number of adverse events possibly correlated and the compliance rate. Secondary outcomes were as follows: (a) clinical remission, defined as a total Mayo score (clinical [c]Mayo plus endoscopic [e]Mayo) less than or equal to 2, with no subscore higher than 1, at week 2 and week 6; (b) clinical response, defined as a reduction in the clinical Mayo score of at least 2 points at week 2, week 6, and week 12 compared to baseline values; (c) endoscopic remission, defined as a Mayo score of 0 to 1, at week 12.

### 2.3. FMT Procedure

#### 2.3.1. Donor Selection

Donors were chosen from among the family members or friends of the patients, according to their suggestions. Donor blood samples were tested for hepatitis A, B, and C, antibodies to HIV-1 and -2, Epstein–Barr virus, Treponema pallidum, Strongyloides stercoralis, and Entamoeba histolytica. Blood cell counts and measurements of transaminase, C-reactive protein, albumin, and creatinine analysis were also performed. Feces were tested for C. difficile (culture and toxin), enteric bacteria, protozoa, and helminths of the large and small bowel, vancomycin-resistant Enterococci, methicillin-resistant Staphylococcus aureus, and Gram-negative (multi-drug-resistant) bacteria. Before donation, a further questionnaire was used to screen for any recent acute gastrointestinal illnesses, newly contracted infections, or other situations that could represent a risk for the patients. Each recipient received feces from the same donor.

#### 2.3.2. Stool Preparation and Fecal Infusion

FMT was performed using fresh feces for the infusion, collected by the donor on the day of the infusion, and processed within 6 h of its collection. At the Microbiology division of our hospital, feces were diluted with 300 mL of sterile saline (0.9%). The deriving solution was blended, and the supernatant was strained and poured into a sterile container. The solution was infused during the colonoscopy procedure using 50 mL syringes filled with the FMT solution through the operative channel of the scope in the cecum. The patients were then asked to maintain a supine position for at least 1 h after the procedure to facilitate as much as possible the permanence of the material infused into the proximal portions of the colon. On average, the entire infusion procedure was performed within 10–15 min. Finally, the patients were monitored in the recovery room of the Endoscopy Center for 2 h after the procedures. All patients admitted to the FMT procedure underwent a standard colonoscopy preparation via a low-fiber diet for three days and a split dose of macrogol of 4 L.

### 2.4. Patient Perspectives Assessment

Patients were monitored through the entire trial, and their perspectives were evaluated via a telephone survey (to avoid direct conditioning by the physician) 24 weeks after the procedure. Three principal questions were asked to patients, and for each question, they were required to provide an answer of “yes”, “no”, or “do not know”. The questions were simple and asked in Italian:

Based on your experience and perception of the study treatment:

(A) “Was FMT efficacious?”
(B) “Was FMT well tolerated?”
(C) “Would you be willing to repeat FMT treatment in the future?”

### 2.5. Microbiota Profiling

Stool samples were collected and immediately frozen at −80 °C, until analysis. DNA was extracted from stool samples (200 mg) using a QIAmp Fast DNA stool mini kit (Qiagen, Hilden, Germany). Microbiota profiles were obtained via the amplification of the V3–V4 region (630 bp) of the 16S rRNA gene following the protocol reported in MiSeq rRNA Amplicon Sequencing (Illumina, San Diego, CA, USA) [23]. The sequencing was performed on an Illumina MiSeqTM platform (Illumina, San Diego, CA, USA). Raw sequences were filtered for quality and chimera presence and matched against the Greengenes 13.8, as described in Del Chierico et al., 2021 [24]. The Shannon index was used to perform α-diversity analysis, and the Bray–Curtis algorithm was applied for β-diversity analysis. Compositional analysis was performed at the genus level of taxonomy. Mann–Whitney and Kruskal–Wallis tests were applied on Shannon index values, the Permanova test was used on the Bray–Curtis distance matrices, and DeSeq2 analysis was applied on taxa relative abundance comparisons. Network analysis was performed using the SparrCC algorithm [25]. All microbiota computational analyses were performed using MicrobiomeAnalyst [26,27].

### 2.6. Statistical Analysisis

Descriptive analyses were used to describe and analyze the characteristics of the study populations at baseline and at each timepoint.

Differences between clinical response and clinical remission at week 6 and 24 for the FMT and Infliximab groups were evaluated using the chi-square test.

## 3. Results

**Figure 1:**
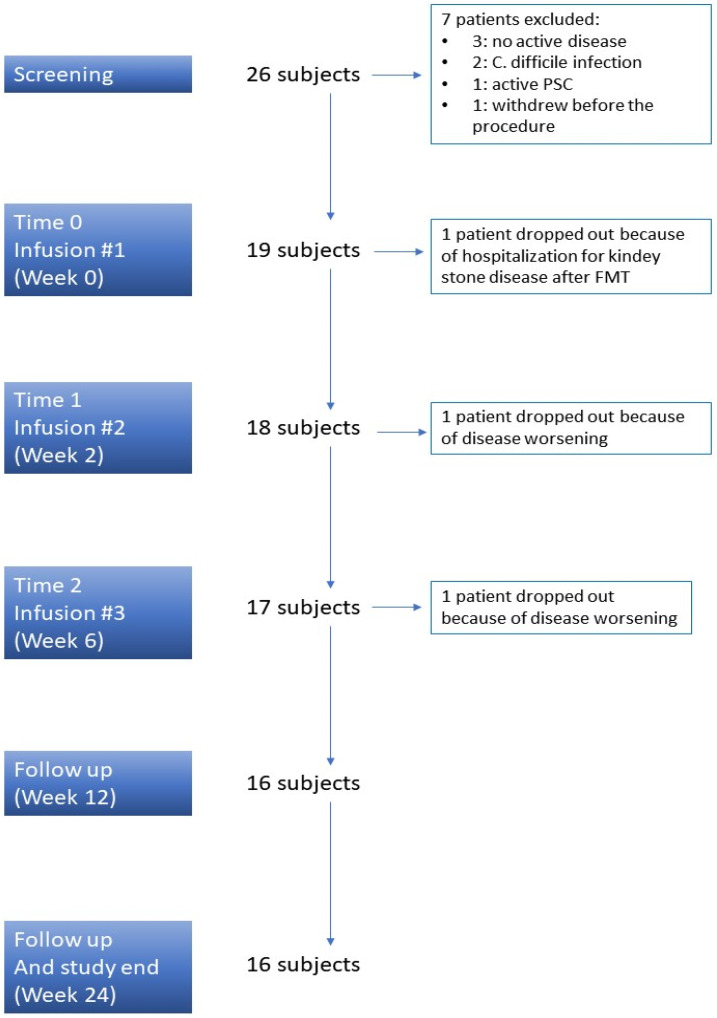
Study design. PSC: Primary Sclerosing Cholangitis.

Overall, 26 consecutive subjects agreed to the study protocol and were considered eligible for inclusion in the FMT protocol. Of them, 19 (73%) were finally enrolled (Figure 1). The clinical characteristics of the included patients are displayed in Table 1.

In total, 19 patients were enrolled in the Infliximab population, whose features are described in Table 2.

### 3.1. Safety Assessment

Only one subject experienced a serious adverse event due to kidney stones after the first fecal infusion and dropped out of the study. Two other patients dropped out due to the disease worsening: one patient required hospitalization to start intravenous steroids at week 2, and the other one required a shift to biologic therapy at week 6 just before the third infusion. Therefore, 16 out of 19 (84%) UC subjects completed the scheduled protocol of three fecal infusions (Figure 1).

After donor feces infusion, 8 of the 16 patients had immediate diarrhea, and 10 experienced bloating and abdominal cramping. In all patients, these symptoms resolved spontaneously within 12–20 h (Table 3).

### 3.2. FMT Perception

**Figure 2:**
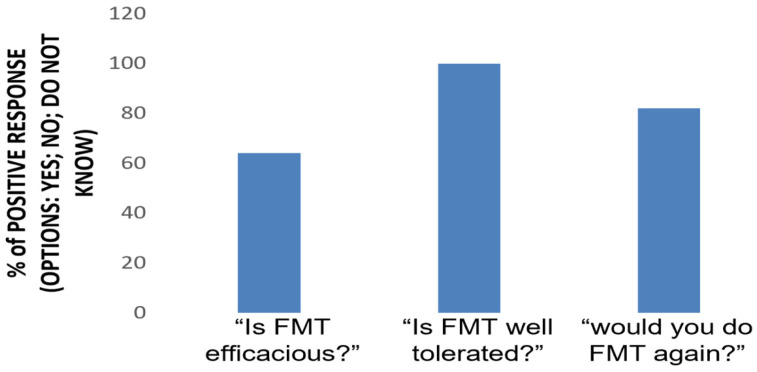
FMT perception. A telephone survey was conducted 24 weeks after the procedure. Three questions were asked to patients, requiring a yes or no answer.

Throughout the entire period of the study, no patient refused to repeat the scheduled colonoscopy at any time, suggesting a good tolerability of the protocol. According to the telephone survey, all patients (100%) considered FMT via colonoscopy to be a well-tolerated procedure without concerns, 82% of them would be willing to repeat it if necessary, and 64% considered FMT to be an effective procedure in the treatment of UC (Figure 2).

### 3.3. Exploratory Efficacy Evaluation

**Figure 3:**
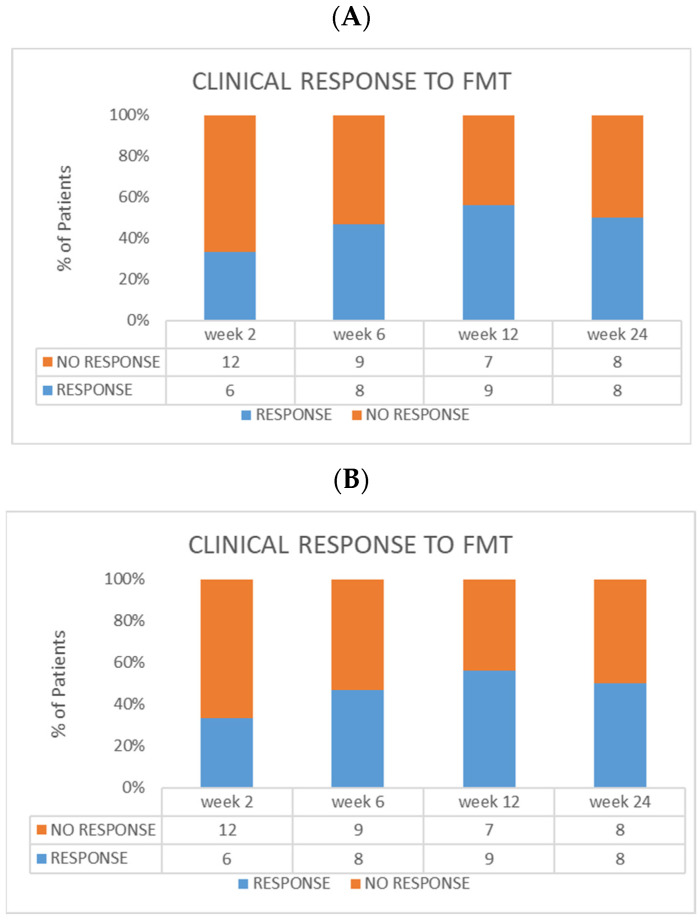
Efficacy evaluation in the FMT group. (**A**) Clinical response at every timepoint. (**B**) Clinical remission at every timepoint. In the same cohort, endoscopic remission at week 12 was reached in six patients (three with Mayo score = 0, and three with score = 1).

A clinical response was obtained in six subjects who completed the scheduled protocol of treatment at week 2, in eight at week 6, and in nine at week 12 (Figure 3A). Clinical remission in the FMT cohort was reached in four subjects at week 2, in five at week 6, and in five at week 12 (Figure 3B). Moreover, clinical response was maintained in eight of the patients from the FMT group until the last follow-up visit at week 24.

Overall, we observed a reduction in the total Mayo score from baseline and throughout the weeks. In particular, we found a reduction in each clinical sub-score (stool frequency, rectal bleeding, and physician assessment) between baseline and week 2, week 6, and week 12.

**Figure 4:**
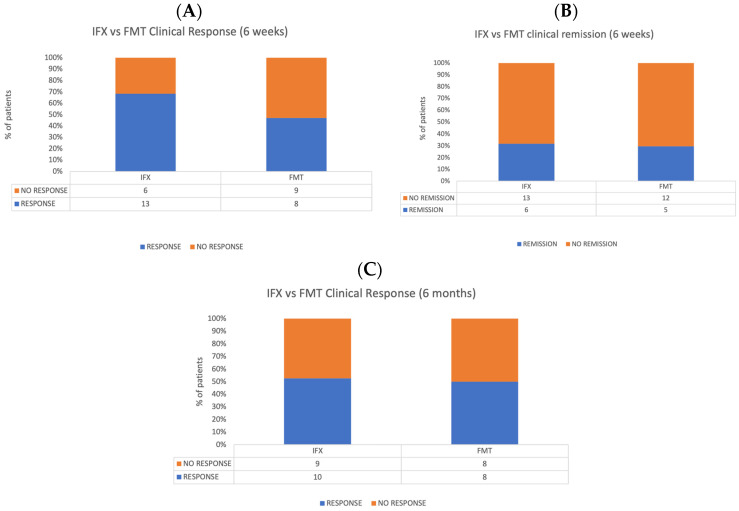
Comparison between FMT and Infliximab populations. (**A**) Clinical response rates at 6 weeks. (**B**) Clinical remission at 6 weeks. (**C**) Clinical response 6 months after the treatment.

In the control population under treatment with Infliximab, clinical response after 6 weeks was achieved in 13 out of 19 patients (68.4%), 6 of which were in clinical remission at this timepoint (31.5%). Ten patients (52.6%) maintained clinical response after 6 months from the beginning of treatment. Chi-squared tests revealed no significant differences between FMT and Infliximab in terms of clinical response and clinical remission at 6 weeks and after 6 months of treatment. Figure 4 shows a comparison between rates of clinical response and remission at different timepoints of the two populations.

### 3.4. Gut Microbiota Foreshadowed the Response to FMT in UC

The microbiota richness was low at T0 and increased with the FMT and during the follow-up.

**Figure 5:**
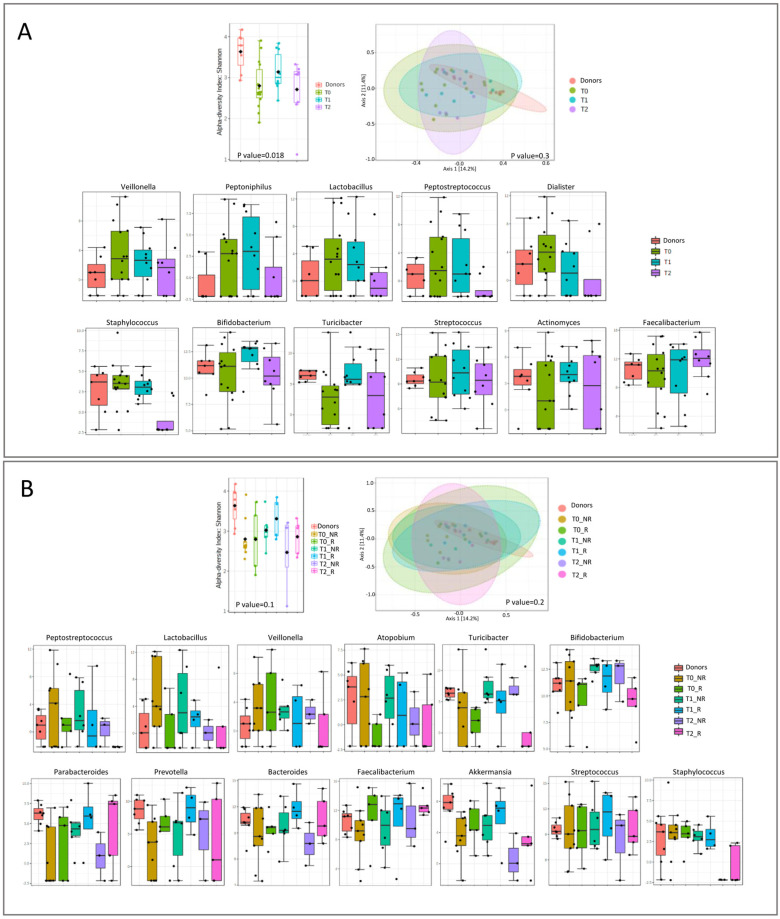
Microbial diversity and taxonomical analysis at T0, T1, and T2: (**A**) overall and (**B**) divided according to responder and non-responder.

*Veillonella*, *Peptoniphilus*, and *Lactobacillus* relative abundances were higher in patients before and immediately after the FMT, compared with the donors. *Peptostreptococcus*, *Dialister*, and *Staphylococcus* showed a marked decrease at T2. At T0, patients and donors showed the same levels of *Bifidobacterium* and *Streptococcus*. After the FMT, these two microorganisms increased at T1 and decreased at T2. *Turicibacter* and *Actinomyces* have a lower abundance at T0 compared with the donors, and it increased at T1 and decreased at T2. The only one that increased during the follow-up and overcame the donor quantity was *Faecalibacterium* (Figure 5A). Grouping the patients into responders and non-responders to FMT, we showed higher bacterial richness in responders at each point of follow-up, compared with non-responders (Figure 5B). *Peptostreptococcus*, *Lactobacillus*, and *Veillonella* were always higher in non-responders, though a gradual reduction after FMT was observed. *Atopobium*, *Turicibacter*, and *Bifidobacterium* were always higher in non-responder compared with responder patients. Interestingly, *Parabacteroides*, *Prevotella*, *Bacteroides*, *Faecalibacterium*, and *Akkermansia* were lower in both patient groups at T0 than in donors. However, responder patients had a higher relative abundance of these bacteria (except for *Prevotella*) at each follow-up point than non-responders, and this percentage increased over time. *Parabacteroides* and *Bacteroides* reached the same donor levels at T2, while *Faecalibacterium* exceeded it. Both non-responder and responder T0 patients had the same levels of *Streptococcus* and *Staphylococcus* as donors. The relative abundance of *Streptococcus* increased at T1 in responder patients and returned to donor levels at T2. In both groups of patients, *Staphylococcus* maintained donor levels at T1, then strongly decreased at T2, until it reached minimal levels.

**Figure 6:**
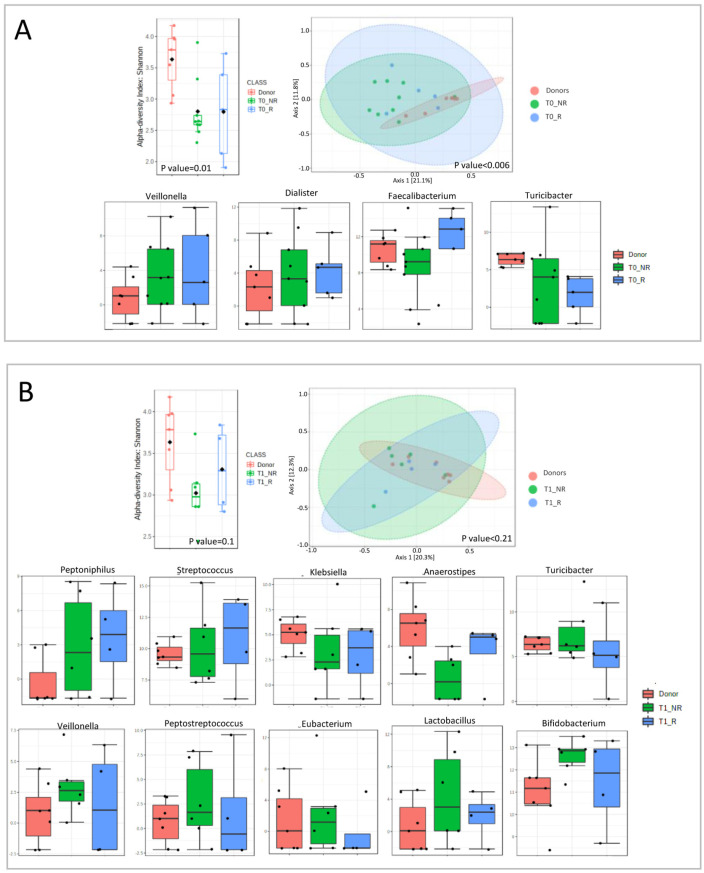

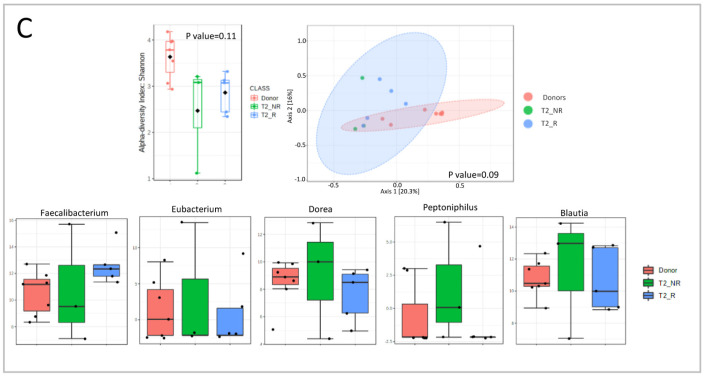
Microbial diversity and taxonomical analysis at each timepoint: (**A**) T0 (first infusion); (**B**) T1 (2 weeks); (**C**) T2 (6 weeks).

Analyzing each timepoint separately, we observed a decrease in bacterial richness for both patient groups at each timepoint (Figure 6).

At T0, we observed an increase in *Veillonella* and *Dialister* for both responders and non-responders compared with donors. *Faecalibacterium* was decreased in non-responders and increased in responders compared with donors. *Turicibacter* was decreased in both groups, compared to the donors, with a major decline in responder patients (Figure 6A).

At T1, *Peptoniphilus* and *Streptococcus* were higher in responder patients, compared with the other two groups. In contrast, *Klebsiella* and *Anaerostipes* were higher in donors than in patients, with a major decline in non-responders. *Turicibacter*, *Veillonella*, *Peptostreptococcus*, *Eubacterium*, *Lactobacillus*, and *Bifidobacterium* were higher in non-responders compared with donors and responders (Figure 6B).

At T2, *Faecalibacterium* was lower in non-responders than in the other two groups. *Dorea*, *Peptoniphilus*, and *Blautia* had the opposite trend, being higher in non-responders compared with donors and responders. *Eubacterium* decreased in both groups of patients (Figure 6C).

**Figure 7:**
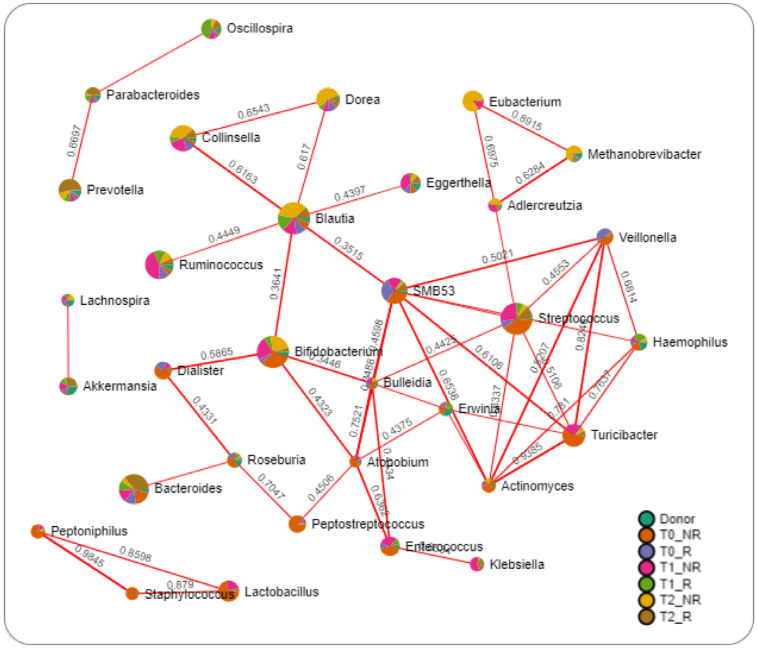
Network analysis of intestinal microbiota using the SparCC algorithm. The nodes represent genera; the edges represent the correlation between genera (red lines, positive correlations). Nodes are colored according to their relative abundance in groups.

Network analysis was performed to reveal relationships between FMT-induced changes in human gut microbiota in responder and non-responder conditions (Figure 7).

The results highlighted only positive relations between bacteria. Interestingly, bacteria such as *Prevotella*, *Parabacteroides*, and *Oscillospira*, which were increased in responder patients, were interconnected. In contrast, *Blautia* linked together with *Dorea*, *Collinsella*, *Ruminococcus*, *Bifidobacterium*, SMB53, and *Eggerthella* were all increased in non-responder time groups. The same can be described for *Eubacterium*, *Methanobrevibacter*, and *Adlercreutzia*, which were all increased in non-responder groups and connected together.

## 4. Discussion

Gut microbiota transplantation is an experimental therapeutic regime in ulcerative colitis, and previously performed randomized controlled trials (RCTs) are, in general, small and methodologically heterogeneous. Furthermore, none of them tested serial fecal infusions as a therapeutic protocol. Furthermore, data comparing FMT to standard-of-care therapies are lacking [28].

This pilot study reports results from the use of serial fecal infusions to induce remission in mildly-to-moderately active UC. The overall feasibility of the study protocol, including serial colonoscopies to release fecal infusions, was largely demonstrated, as all patients concluded the trial without any major adverse events related to the procedure. In addition, all patients underwent the foreseen procedures. In particular, three colonoscopies were well tolerated and accepted by the patients, and procedures were successful without any noteworthy side effects, as well underlined by positive scores of patients rating their experience with FMT at the end of the trial via a phone-call-based questionnaire.

This is the first trial using three consecutive fecal infusions via colonoscopy over 6 weeks (at time 0, after 2 weeks, and after 6 weeks) as a scheme of induction therapy for UC. From this perspective, our trial, despite coming after the three RCTs already published, is still adding data to the current knowledge on FMT. In fact, while different routes of delivery do not appear to considerably modify the efficacy rates of FMT in *C. difficile* infection, our trial is pushing the overall efficacy toward more positive results when FMT is performed via colonoscopy.

The response rate achieved via repeated colonoscopy infusions of fecal microbiota was much higher than those obtained in the RCTs by Moayyedi and Rossen [17,27]. This finding should not be considered as an “out of the box” result, as the lower route may achieve higher efficacy rates than the upper one, perhaps by preserving microbiota via potential modification throughout the small intestine.

From this perspective, it should not be surprising that the two more similar trials (ours and the one by Moayyedi P et al.) [17] display positive results compared with the trial proposed by Rossen et al. [22].

The clinical remission obtained in the present study was comparable to Paramsothy’s study (31% both at week 6 and at week 12 in our study vs. 27% at week 8 in Paramsothy’s study) [19]. Obviously, the results of these two studies refer to different settings, but it is worth pointing out that both provided intensive fecal infusions, with the only difference being that the infusions that followed the first were carried out via enemas in Paramsothy’s study. Serious and mild adverse events were similar as well.

Another important finding of our study was that mucosal healing was obtained in 6 subjects. These results are important not only to consider FMT as a possible therapeutic option in well selected patients (those with mildly-to-moderately active UC) but also to propose colonoscopy as a way of infusion, in order to increase the chances for a successful and sustained recipient-donor microbiota engraftment.

Interesting considerations can be put forward by observing the comparison between the FMT cohort and the control population under treatment with Infliximab, the progenitor of anti-TNFα drugs, and biologic medications. In fact, at 6 weeks from baseline, the Infliximab cohort shows a higher rate of response than the FMT population; nonetheless, the rates of response of the two populations tend to equalize after 6 months from baseline. Taking into consideration that patients were allowed to take other medications during our trial, this finding can be explained by considering FMT and gut microbiota restoration as enhancers of other therapeutic options, with a predominantly long-term action. Although our trial was not designed to show equivalence or superiority among the two treatment modalities, the similar results displayed by using FMT and IFX in this category of patients suggest the importance of patient selection to maximize response rate, rather than the superiority per se of a treatment option compared to another.

The gut microbiota profiling highlighted the role in predicting the negative FMT response of some bacteria such as *Peptostreptococcus*, *Lactobacillus*, and *Veillonella*, which were higher in non-responders in comparison to donors and responders. In contrast, *Parabacteroides*, *Bacteroides*, *Faecalibacterium*, and *Akkermansia* were increased in responder patients during the study period, indicating a positive and active role in FMT response.

The microbial analysis performed in our FMT population confirms the hypothesized mechanism of FMT in UC. In fact, FMT favors the restoration of a healthy and diverse microbial community within the recipient’s gut by introducing a diverse range of beneficial microorganisms. The transplanted fecal material contains a complex mixture of bacteria, viruses, fungi, and other microorganisms that collectively contribute to the restoration of a more balanced gut microbiota. These microorganisms may help modulate the recipient’s immune response, reduce inflammation, enhance the production of short-chain fatty acids (SCFAs), and promote mucosal healing in the colon.

Furthermore, FMT may also influence the recipient’s immune system through various mechanisms, including the transfer of regulatory T cells, modulation of dendritic cell function, and alteration of cytokine profiles. These immune-related effects can contribute to the downregulation of the inflammatory response associated with UC.

Overall, FMT via colonoscopy in patients with UC is believed to exert its therapeutic effects through the restoration of a healthier gut microbiota composition, modulation of the immune response, and promotion of mucosal healing [29].

Our findings reveal an intriguing association between a higher abundance of *Fecalibacterium prausnitzii* and favorable treatment responses in our fecal microbiota transplantation (FMT) trial. This aligns with several previous studies both in the field of IBD [30] and Clostridioides difficile diarrhea [31].

*F. prausnitzii* is considered to be a beneficial bacterium primarily due to its role in maintaining gut health and homeostasis. This species is a prominent producer of short-chain fatty acids (SCFAs), particularly butyrate, which serves as a crucial energy source for colonic epithelial cells and exerts anti-inflammatory effects. Butyrate production by *F. prausnitzii* has been associated with the reinforcement of the intestinal barrier function and the modulation of the immune response, both of which are essential for mitigating inflammation and promoting gastrointestinal well-being [32].

Our study has also uncovered a noteworthy correlation between higher levels of Akkermansia and enhanced response rates to fecal microbiota transplantation (FMT) among our patient cohort. *Akkermansia muciniphila*, a prominent member of the gut microbiota, has garnered increasing attention as a potential beneficial bacterium in various gastrointestinal disorders. One of its notable attributes is its capacity to degrade mucin, the glycoprotein that lines the intestinal epithelium. By doing so, *Akkermansia* plays a role in maintaining mucosal integrity and bolstering the gut barrier function, thus potentially reducing susceptibility to gut inflammation and permeability. Moreover, *Akkermansia* has been associated with improved metabolic parameters and anti-inflammatory effects, which further supports its status as a potentially beneficial microbe [33].

The potential significance of *Akkermansia* as a microbial marker in UC has been investigated in preclinical studies as well. In fact, it has been shown that Akkermansia reduced the severity of colitis in DSS murine models [34]. The mechanisms underpinning *Akkermansia*’s contributions to therapeutic responses warrant further investigation. Potential avenues include its ability to enhance mucosal health, influence the gut–immune axis, and modulate metabolic functions.

In addition, our study has unveiled a notable contrast in the gut microbiota composition of non-responders to FMT, characterized by elevated levels of *Peptostreptococcus*, *Lactobacillus*, and *Veillonella*.

An increased abundance of *Peptostreptococcus* was observed in previous studies in which higher concentrations of this bacteria were associated with both inflammatory bowel disease and colorectal cancer [35,36].

Elevated levels of *Lactobacillus* in non-responders to FMT resonate with studies implicating certain Lactobacillus strains in promoting intestinal inflammation and diminishing treatment efficacy. While *Lactobacillus* is generally associated with beneficial effects regarding gut health, it is important to note that the genus Lactobacillus encompasses a diverse group of species, some of which may have differing effects in the context of UC and FMT [37].

The increased prevalence of *Veillonella* in non-responder patients is in line with research highlighting, for instance, in a pediatric multicentric population, how the abundance of *Veillonella* was associated with an increased risk of penetrating complications [38].

Nevertheless, our paper has limitations. First, we did not collect the fecal samples from all patients for microbiota assessment, as they were not fully available for the present publication. We also believe that being an exploratory feasibility trial, microbial data could have been difficult to analyze and interpret for such a small number of patients. In addition, in the present paper, we analyzed only the induction of remission and clinical response after FMT, without a real “maintenance” strategy or a “re-do trial” strategy. However, our objective was to investigate the feasibility and safety of a protocol including intensive serial colonoscopies as the route of administration of fecal infusions in UC patients. We are aware that this is only a pilot study that may chart a course for future RCTs, including different methods of infusion and a placebo treatment group.

## 5. Conclusions

Our study suggests that FMT via colonoscopy may be a reliable option for inducing remission in UC, although other less invasive strategies—perhaps enemas—should be proposed for maintenance. Overall, this trial reinforces the positive results reported in other studies [17,19], highlighting colonoscopy as a feasible and potentially effective approach to curing patients with UC through the use of FMT, at least in an induction phase. Although many questions remain, our results support the idea that active modulation of the microbiota could represent a reliable and promising therapeutic avenue for managing UC [2].

## Figures and Tables

**Table 1:** Clinical characteristics of the enrolled FMT patients.

Characteristics	Study Population
**Sex**	
Males	11
Females	8
**Mean age (years)**	44.7
**Type of disease**	
-Left colitis	13
-Pancolitis	6
**Mean disease duration (years)**	6.75
**Previous biologic therapy**	
Yes	9
No	10
**Concomitant oral mesalazine**	
Yes	15
No	4
**Concomitant oral steroids**	
Yes	8
No	11
**Concomitant topical therapy**	
Yes	6
No	13
**Concomitant immunosuppressant drugs**	
Yes	1
No	18
**Clinical disease activity (Mayo Score)**	
-Remission (0–1)	0
-Mild–moderate (2–4)	6
-Moderate–severe (5–7)	13
-Severe (8–9)	0

**Table 2:** Clinical characteristics of the enrolled Infliximab patients.

Characteristics	Infliximab (IFX) Population
**Sex**	
M	8
F	11
**Mean age (years)**	36.6 yrs
**Type of disease**	
-Left colitis	9
-Pancolitis	10
**Mean disease duration (years)**	7.9 yrs
**Previous biologic therapies**	
Yes	5
No	14
**Clinical disease activity (Clinical Mayo score)**	
-Average	5.7
-Remission (0–1)	0
-Mild–moderate (2–4)	6
-Moderate–severe (5–7)	8
-Severe (8–9)	5

**Table 3:** Safety assessment in enrolled FMT patients. FMT = fecal microbiota transplantation; SAE = severe adverse events.

	FMT (19 cases)
SAE	1 (5%) Hospitalization for kidney stone disease
Disease worsening	2 (10%)
Infusion reaction	0
Total	3 (15%)

## Data Availability

Full data are unavailable due to privacy or ethical restrictions; however, authors can ask permission following specific requests.
